# The Potential Antinociceptive Effect and Mechanism of *Cannabis sativa* L. Extract on Paclitaxel-Induced Neuropathic Pain in Rats Uncovered by Multi-Omics Analysis

**DOI:** 10.3390/molecules29091958

**Published:** 2024-04-25

**Authors:** Yunhui Xu, Lijuan Yao, Yuhan Guo, Chenfeng Shi, Jing Zhou, Moli Hua

**Affiliations:** National Key Laboratory of Lead Druggability Research, Shanghai Institute of Pharmaceutical Industry Co., Ltd., China State Institute of Pharmaceutical Industry, Shanghai 201203, China; xuyh1017@126.com (Y.X.); 18162457868@163.com (L.Y.); gyh_syphu189@163.com (Y.G.); scf417586179@163.com (C.S.); jingzs427@163.com (J.Z.)

**Keywords:** hemp, transcriptome, metabolome, gut microbiota, paclitaxel-induced peripheral neuropathy, supercritical carbon dioxide extraction, analgesic mechanism

## Abstract

*Cannabis sativa* L. (hemp) is a herbaceous plant rich in cannabinoids with a long history of use in pain treatment. The most well-characterized cannabinoids, cannabidiol (CBD) and Δ9-tetrahydrocannabinol (Δ9-THC), garnered much attention in chemotherapy-induced peripheral neuropathy (CIPN) treatment. However, few studies have investigated the biological benefits and mechanism of hemp extract on CIPN. In the present study, hemp extract (JG) rich in cannabinoids was extracted by supercritical fluid carbon dioxide extraction (SFCE). The antinociceptive efficacy was evaluated using a paclitaxel-induced peripheral neuropathy (PIPN) rat model based on behavioral tests. Further omics-based approaches were applied to explore the potential mechanisms. The results showed that JG decreased mechanical allodynia, thermal hyperalgesia, and inflammatory cytokines in PIPN rats significantly. Transcriptome analysis identified seven key genes significantly regulated by JG in PIPN model rats, mainly related to the neuroactive ligand–receptor interaction pathway, PPAR signaling pathway, and cAMP signaling pathway. In metabolomic analysis, a total of 39 significantly altered metabolites were identified, mainly correlated with pentose and glucuronate interconversions and the glycerophospholipid metabolism pathway. Gut microbiota analysis suggested that increased community *Lachnoclostridium* and *Lachnospiraceae_UCG-006* in PIPN rats can be reversed significantly by JG. In conclusion, hemp extract exhibited antinociceptive effects on PIPN. The analgesic mechanism was probably related to the regulation of inflammation, neuroactive ligand–receptor interaction pathway, sphingolipid metabolism, etc. This study provides novel insights into the functional interactions of *Cannabis sativa* L. extract on PIPN.

## 1. Introduction

*Cannabis sativa* L., commonly known as industrial hemp, is a herbaceous plant that belongs to the family Cannabinaceae. Hemp produces a diverse array of secondary metabolites, including phytocannabinoids, terpenes, and phenolic compounds with potential pharmaceutical values as drugs or supplements [1]. The most well-characterized phytocannabinoids are cannabidiol (CBD), Δ-9-tetrahydrocannabinol (Δ9-THC), cannabidivarin (CBDV), cannabinol (CBN), cannabidiolic acid (CBDA), cannabigerol (CBG), cannabichromene (CBC), etc. [2]. They have garnered much attention of late for the treatment of various conditions associated with pain and inflammation [3].

Chemotherapy-induced peripheral neuropathy (CIPN) is a cumulative, dose-dependent adverse event and is associated with some of the most commonly used chemotherapeutic agents, including platinum agents, taxanes, vinca alkaloids, bortezomib, and thalidomide [4]. Possible mechanisms of CIPN development may be involved in disrupted microtubule-mediated axonal transport, axonal degeneration, direct damage to the dorsal root ganglion, and mitochondrial dysfunction [5]. But, the course of CIPN is unpredictable and varies with each of the several chemotherapeutic agents, reflecting their distinctive mechanism of action [4,6].

Paclitaxel (PTX), belonging to the taxanes group, is widely employed alone or combined with other chemotherapeutic agents against many types of tumors. PTX binds to and stabilizes microtubules, interrupting mitosis in the G2/M phase, followed by cell death and apoptosis [7]. However, they cause peripheral neuropathy as an adverse event. In paclitaxel-induced peripheral neuropathy (PIPN), many patients develop sensory abnormalities such as numbness, pain, and burning sensation in the hands and feet [8]. This neuropathic pain is considered particularly difficult to treat. Symptoms in some people can linger for up to 3 years or last a lifetime after the tumor therapy stops, which seriously impacts the quality of life [9]. However, the clinical treatments, including primarily duloxetine, gabapentin, pregabalin, and amitriptyline, have shown limited efficacy and many adverse reactions, such as somnolence, dizziness, and addiction [10]. As a result, there is an urgent need to find a therapy that effectively manages PIPN.

Several reports can be found to explore the antinociceptive effect of phytocannabinoids in the CIPN model [11,12]. Most of them focus on the two major constituents, Δ9-THC and CBD. CBD may prevent the development of paclitaxel-induced allodynia, and the effect can be reversed by co-administration of the 5-HT 1A antagonist [13,14]. Inhaled vaporized cannabis (containing 10.3% Δ9-THC/0.05% CBD) produced antinociception in paclitaxel-treated rats [15]. Kumar Kalvala A investigated the neuroprotective effects of synthetic CBD and tetrahydrocannabivarin (THCV) and their combination on PIPN in mice, and the entourage effect of CBD and THCV combination against PIPN appears to protect neurons in mice by modulating 5HT1A and CB1 receptors, respectively. The transcriptome changes related to CBD and THCV against PTX-induced neuropathy were evaluated to explore the related mechanism [16]. King KM reported the various efficacies of CBD and THC on different CIPN models. Both CBD and THC alone attenuated mechanical allodynia in mice treated with paclitaxel. CBD also attenuated oxaliplatin- but not vincristine-induced mechanical sensitivity, while THC significantly attenuated vincristine- but not oxaliplatin-induced mechanical sensitivity [17].

As for other forms of neuropathic pain, the antinociceptive effect of full-spectrum cannabis extract containing 64.5% CBD, 4% THC, and <4% other cannabinoids has been confirmed in streptozotocin-induced diabetic neuropathy and chronic constriction injury of the sciatic nerve (CCI) model. In this study, full-spectrum cannabis extract has a better analgesic effectiveness than CBD or THC alone, and the antinociceptive effect was independent of CB1 and CB2 receptors, mainly due to the activation of the vanilloid receptor, TRPV1 [18,19]. However, few studies have investigated the biological benefits and mechanism of hemp extract on PIPN.

Cannabinoids from hemp can be extracted using various extraction processes, such as solvent infusion, microwave-assisted extraction, and supercritical fluid extraction (SFE). SFE is one of the green and efficient extraction techniques for medium-polarity compounds. The most favorable solvent used in SFE is carbon dioxide (CO2) because of its low cost, abundance, and valorization potential. It has been used to extract secondary metabolites from hemp successfully [20,21].

Omics-based approaches, including genomics, transcriptomics, proteomics, metabolomics, etc., have become recognized as effective tools enabling researchers to better understand disease status and discover natural product targets [22]. Previous related works barely use similar technology to comprehensively analyze the action mechanisms of *Cannabis sativa* L.

The objective of this study was to assess the antinociceptive effect of CBD-enriched hemp extract on the PIPN rat model by changes in behavior tests and inflammatory factors and investigate the molecular mechanism based on transcriptomic, metabonomic, and gut microbiota analysis.

## 2. Results

### 2.1. Chemical Composition of Hemp Extract (JG)

Phytocannabinoids are oxygen-containing C 21 aromatic hydrocarbons found in hemp, including neutral cannabinoids and acidic cannabinoids. Acidic cannabinoids in dried cannabis generally carry a carboxylic acid moiety and undergo spontaneous loss of this carboxyl moiety when subjected to high temperatures. Supercritical fluid carbon dioxide extraction (SFCE) is widely used for cannabinoid extraction [20]. In this study, the Supercritical CO_2_ extractives of hemp were collected from the separation vessel with a yield of 4.6% (*w*/*w*). The crude extract was subjected to heat (125 °C for 5 h) for decarboxylation, and 85% ethanol precipitation was processed to separate chlorophyll, waxes, and other impurities to obtain the final cannabis extract (JG). Subsequently, the chemical composition of JG was analyzed by using UPLC-QTOF/MS in both positive and negative ion modes. The base peak intensity chromatogram (BPI) is shown in Supplementary Materials Figure S1. A total of 33 compounds were identified by tandem mass spectrometry (Table 1), and the most abundant compound, CBD, shows a content of 30.10% tested by HPLC. Meanwhile, the content of other normal phytocannabinoids was calculated by relative peak area based on CBD, which is exhibited in Table 2.

### 2.2. Antinociceptive Effect of Hemp Extract (JG) on PTX-Induced Neuropathic Rats

The antinociceptive effect was evaluated by using a PIPN rat model. In this experiment, PTX acted as a model inducer in normal rats. Gabapentin is a clinically used drug for the treatment of neuropathic pain performed as a positive drug control. The analgesic effect was evaluated based on behavioral tests and inflammatory cytokines.

#### 2.2.1. Effect of Hemp Extract (JG) on Neurobehavioral Changes of PTX-Induced Neuropathic Rats

**Figure 1:**
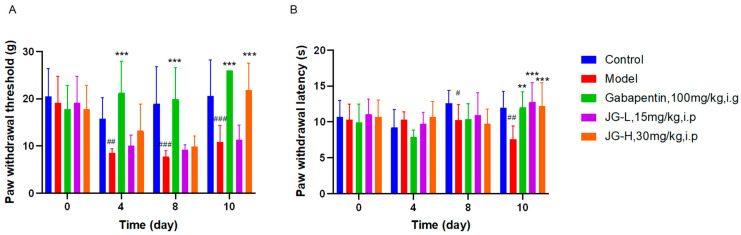
Hemp extract (JG) treatment reversed the reduced mechanical and thermal allodynia in the PTX-induced neuropathic rats. (**A**) Paw withdrawal threshold for mechanical allodynia evaluated by the Von Frey test in rats (*n* = 8; mean ± SD); (**B**) paw withdrawal latency evaluated by thermal stimulation in rats (*n* = 8; mean ± SD); (Control—blank control; Model—rats treated with paclitaxel 2 mg/kg daily on days 1, 3, 5, and 7 i.p. to build neuropathic model, Gabapentin-positive drug. JG—hemp extract, PTX—paclitaxel. Data were analyzed by one-way ANOVA. # *p* < 0.05, ## *p* < 0.01, ### *p* < 0.001 vs. Control group; ** *p* < 0.01, ***p < 0.001 vs. Model group).

Compared with the Control group, the mechanical threshold (Figure 1A) and thermal threshold (Figure 1B) of the Model group (PTX-induced neuropathic rats) from day 4 and day 8, respectively, were significantly declined (*p* < 0.05), which indicated that the neuropathic model was successfully established. Compared with the Model group, the mechanical threshold of the positive drug Gabapentin group and hemp extract JG-H group (30 mg/kg) increased significantly from day 4 and day 10, respectively (*p* < 0.05). But, the JG-L group (15 mg/kg) did not show a significant difference (Figure 1A). The result indicated a dose-dependent effect of JG on the rising mechanical threshold. Compared with the Model group, the thermal threshold revealed a significant increase in the Gabapentin and JG groups on day 10 (*p* < 0.05) (Figure 1B). The results above revealed a significant effect of JG in the treatment of neuropathic pain.

#### 2.2.2. Effect of Hemp Extract (JG) on Inflammatory Cytokines of PTX-Induced Neuropathic Rats

The literature has shown that chemotherapy directly results in an upregulation of various well-known pro-inflammatory mediators, such as IL-1β, TNF-α, MCP-1, IL-6, etc., which contribute to symptoms including but not limited to fatigue, peripheral neuropathy, and cognitive decline [25]. So, we detected the effects of JG on inflammatory cytokines by ELISA in PTX-induced neuropathic rats.

**Figure 2:**
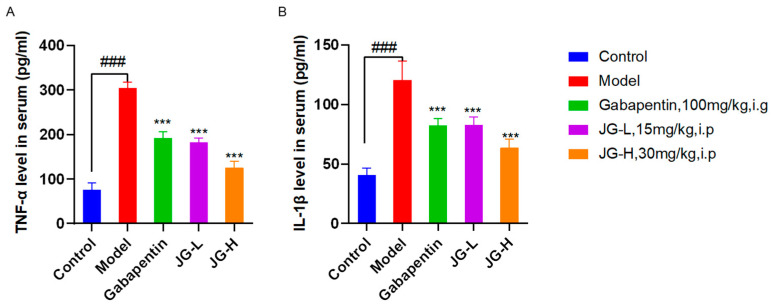
JG treatment improved inflammatory cytokines in the PTX-induced neuropathic rats. Serum level of inflammatory cytokines TNF-α (**A**) and IL-1β (**B**) tested by ELISA in rats (*n* = 8; mean ± SD) (Control—blank control; Model—rats treated with paclitaxel 2 mg/kg daily on days 1, 3, 5, and 7 i.p. to build neuropathic model, Gabapentin-positive drug. JG—hemp extract, PTX—paclitaxel. Data were analyzed by one-way ANOVA. ### *p* < 0.001 vs. Control group; ***p < 0.001 vs. Model group).

Compared with the Control group, TNF-α and IL-1β levels in the serum of the Model group rats were significantly increased (*p* < 0.05) (Figure 2), which indicated that the inflammatory cytokines changed in the PTX-induced neuropathic rats. Compared to the Model group, Gabapentin and JG treatment reduced the levels of inflammatory cytokines significantly (*p* < 0.05).

### 2.3. Transcriptome Analysis

In order to clarify the mechanism of PTX-induced neuropathic pain and the antinociceptive mechanism of JG, we performed transcriptome sequencing analysis. After Novaseq sequencing, raw reads were obtained from 12 spinal cord samples of the Control, Model, and JG-H groups. After quality control checks, clean reads were obtained. The clean reads with Q20 > 98.2%, Q30 > 94.8%, and a GC content of 47.7% (Table S1) were aligned and mapped to the reference genome of Rattus norvegicus genome (version Rnor_6.0) using Hisat2 (v2.2.1). The results showed that, on average, 95.7% of the reads were successfully mapped to the reference genome.

#### 2.3.1. Identification of Differentially Expressed Genes (DEGs)

**Figure 3:**
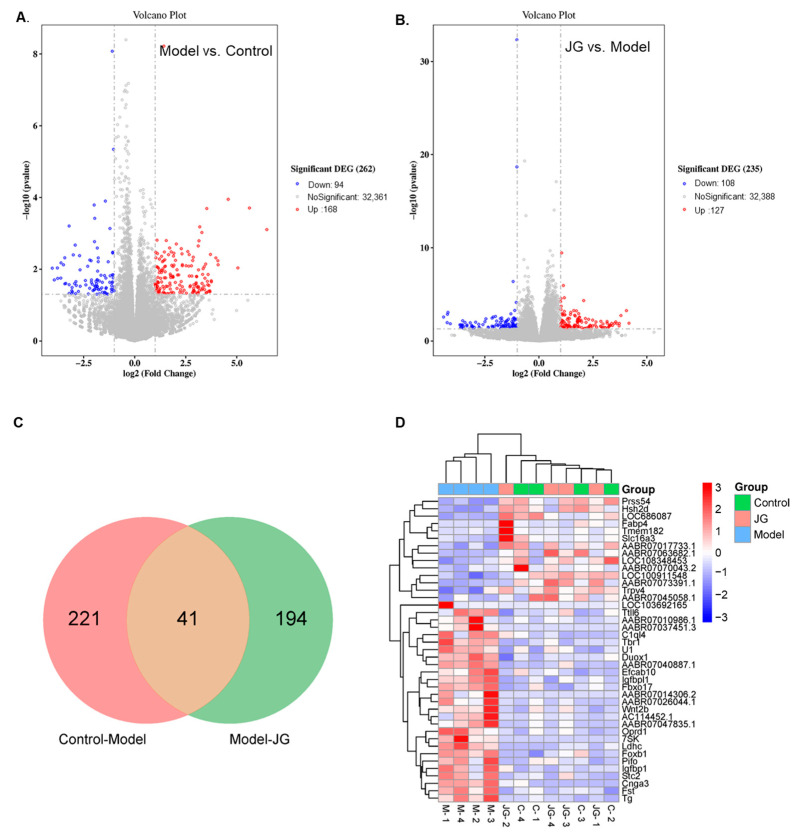
Differential expression genes (DEGs) filtered by *p* < 0.05 and log 2 (fold change) > 1. (**A**) The volcano diagram of DEGs between the Model group and Control group. (**B**) The volcano diagram of DEGs between the JG group and Model group. Red plots represent up-regulated genes, and blue plots represent down-regulated genes. (**C**) A total of 41 common DEGs between Model-DEGs and JG-DEGs. (**D**) The clustering Heatmap of 41 common DEGs. The *x*-axis represents the samples from the Control (C), Model (M), and JG groups, and the *y*-axis represents different genes; red means genes exhibiting increased expression, and blue means genes exhibiting decreased expression.

Based on the threshold log_2_ (fold change) > 1, *p* < 0.05, differential expression genes (DEGs) were filtered using the DESeq2 Bioconductor package. Compared with the Control group, 168 DEGs were up-regulated and 94 DEGs were down-regulated in the Model group, recorded as Model-DEGs (Figure 3A). Meanwhile, 127 DEGs were up-regulated and 108 DEGs were down-regulated in the JG group compared with the Model group, recorded as JG-DEGs (Figure 3B). Moreover, there are 41 common DEGs between the Model-DEGs and JG-DEGs, which may be directly related to the antinociceptive effect of JG (Figure 3C, Table 3). The corresponding heatmap provides a visual representation of the changing trends, in which we observed that JG treatment reversed aberrant gene expression profiles associated with PTX treatment (Figure 3D).

#### 2.3.2. Gene Ontology (GO) Enrichment and Kyoto Encyclopedia of Genes and Genomes (KEGG) Pathway Analysis of DEGs

**Figure 4:**
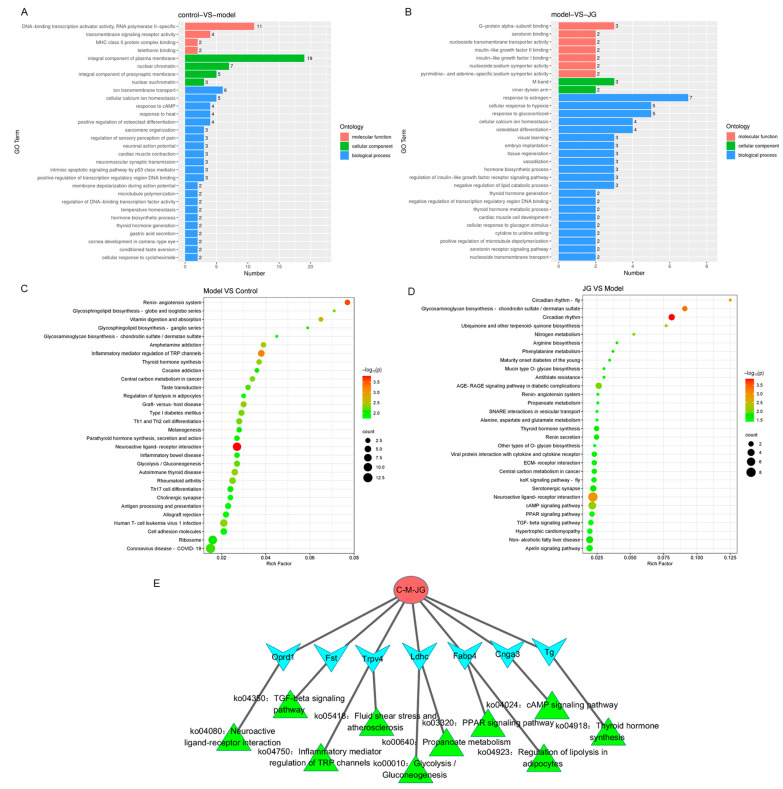
GO enrichment analysis and KEGG pathway enrichment analysis of differentially expressed genes (DEGs). (**A**) The top 30 GO terms annotated by Model-DEGs. (**B**) The top 30 GO terms annotated by JG-DEGs. (**C**) The top 30 KEGG pathways enriched by Model-DEGs. (**D**) The top 30 KEGG pathways enriched by JG-DEGs. (**E**) Core network for common DEGs and pathways indicating the potential antinociceptive mechanism of JG on PIPN rats.

To further explore the biological significance of the differentially expressed genes and the analgesia mechanism of JG, the DEGs were categorized according to GO functional annotation terms and KEGG pathway analysis. A total of 332 GO terms were significantly enriched in Model-DEGs, encompassing 239 terms in biological processes (BPs), 25 terms in cellular components (CCs), and 68 terms in molecular functions (MFs). The top 30 GO items are shown in Figure 4A. Among the GO terms, the classification of BPs demonstrated that the DEGs were mainly involved in ion transmembrane transport, cellular calcium ion homeostasis, response to cAMP, response to heat, temperature homeostasis, osteoclast differentiation, etc. Classification of the CC showed that the DEGs were mainly distributed in the presynaptic membrane, plasma membrane, and nuclear euchromatin. The MF classification results showed that the main molecular functions of DEGs were associated with DNA-binding transcription activator activity and transmembrane signaling receptor activity. While in JG-DEGs, a total of 397 GO terms were significantly enriched, with 286 terms in BPs, 28 terms in CCs, and 83 terms in MFs. The top 30 GO items are shown in Figure 4B. Classification of BPs demonstrated that the DEGs were mainly involved in response to estrogen, response to glucocorticoid, cellular calcium ion homeostasis, osteoblast differentiation, lipid catabolic process, and serotonin receptor signaling pathway. Classification of MF results showed that the main molecular functions of DEGs were associated with G-protein alpha-subunit binding, serotonin binding, nucleoside transmembrane transporter activity, and sodium symporter activity.

KEGG enrichment analysis of DEGs showed that transcriptome change in the PIPN model is mainly involved in pathways of neuroactive ligand–receptor interaction, inflammatory mediator regulation of TRP channels, and the renin–angiotensin system. At the same time, JG treatment may play a role in the analgesic effect by regulating neuroactive ligand–receptor interaction, circadian rhythm, cAMP signaling pathway, and PPAR signaling pathway. The top 30 terms of enriched pathways are shown in Figure 4C,D. According to the common DEGs (eliminating those nonhuman genes, 39 DEGs included) between Model-DEGs and JG-DEGs, a core network including 7 DEGs associated with 10 enriched pathways (significantly enriched pathways of Model-DEGs or JG-DEGs, *p* < 0.05) was extracted, as shown in Figure 4E. These findings provide new insights into the potential mechanisms underlying the analgesia properties of JG in PIPN rats.

### 2.4. Untargeted Metabolome Analysis

#### 2.4.1. Identification of Differential Metabolites (DMs)

The metabolic profile of rat serum in the Control, Model, and JG groups was detected by UPLC-Q-TOF/MS, and the total ion chromatogram (TIC) was collected by MassLynxV4.1(SCN937). Figure S2 presents their base peak ion (BPI) chromatogram.

**Figure 5:**
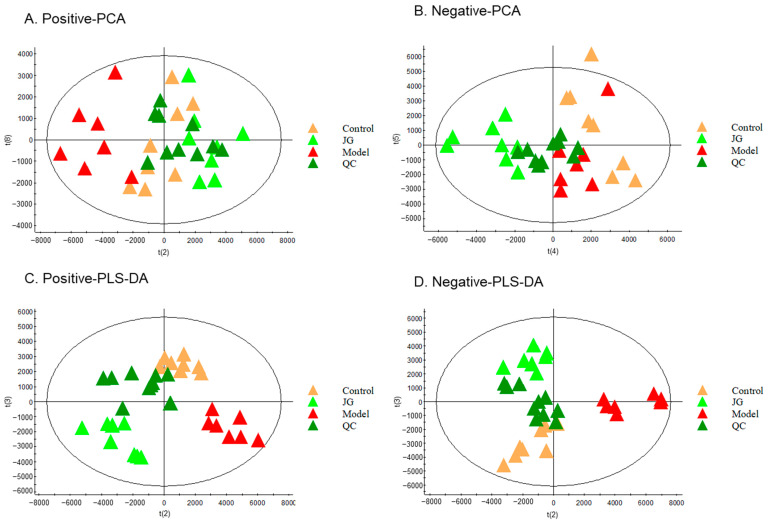
Score scatter plot for PCA and PLS-DA analysis for rat serum metabolomic analysis among the Control, Model, and JG groups. (**A**,**C**) Positive mode (R2Y = 91%, Q2 = 75% for PLS-DA). (**B**,**D**) Negative mode (R2Y = 81%, Q2 = 68% for PLS-DA).

To understand the overall metabolic difference and the variability between the samples in the group, we conducted unsupervised principal component analysis (PCA) and supervised partial least squares discriminant analysis (PLS-DA). Figure 5 demonstrates the aggregation in the QC samples observed from the PCA well. Therefore, it can be concluded with certainty that the LC-MS system maintained suitable stability throughout the analysis. Figure 5 also shows that there was a tendency for separation between the groups among the Control, Model, and JG groups, as well as a very apparent aggregation tendency within each group.

**Figure 6:**
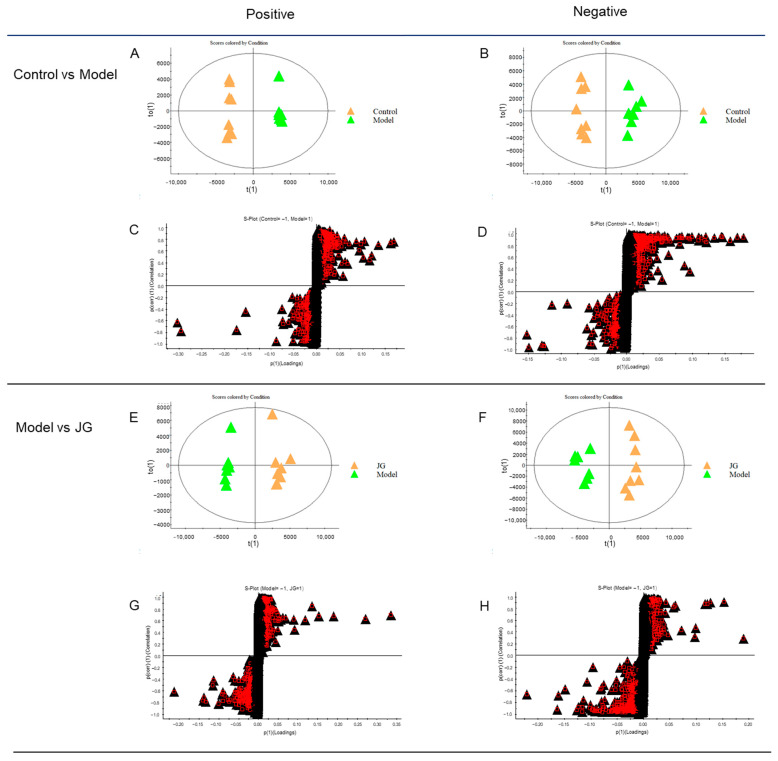
Score scatter plots for OPLS-DA analysis and their corresponding S-plot for rat serum metabolomic analysis among the Control, Model, and JG groups. (**A**,**C**) Control vs. Model in positive mode (R2Y = 99%, Q2 = 89%). (**B**,**D**) Control vs. Model in negative mode (R2Y = 97%, Q2 = 90%). (**E**,**G**) JG vs. Model in positive mode (R2Y = 97%, Q2 = 90%). (**F**,**H**) JG vs. Model in negative mode (R2Y = 95%, Q2 = 91%). Black triangles represent metabolites, triangles with red box represent metabolites with VIP > 1 in (**C**,**D**,**G**,**H**).

The discriminant analysis of orthogonal correct partial least squares method (OPLS-DA) shown in Figure 6A,B,E,F reveals a significant inter-group difference and intra-group aggregation. The S-plot and variable importance in projection (VIP) were used to identify potential different metabolites (DMs). Figure 6C,D,G,H show the S-plot, which displays the ions contributing to the separation of the two groups. Each ion is represented as a point on the S-plot, and the VIP value indicates the significance of the ion in the differentiation of the two groups; the greater VIP represents a larger ion differentiation between the two groups.

**Figure 7:**
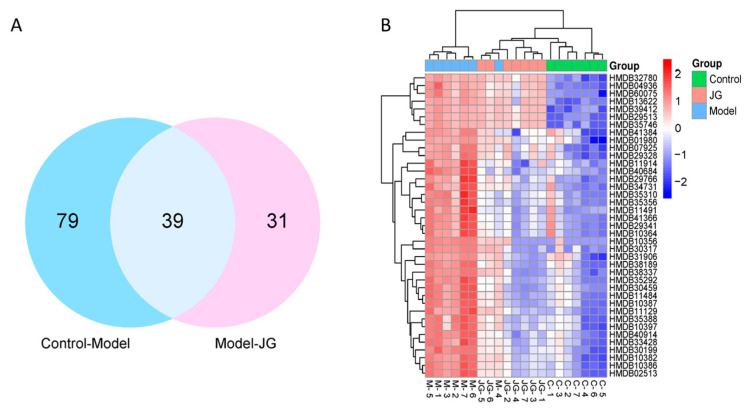
Common differential metabolites (DMs) among the Control, Model, and JG groups. (**A**) Venn diagram showing common DMs that appeared in each group. (**B**) Heatmap showing the differences in expression of the DMs in the three groups. Red and blue colors indicate higher or lower expression, respectively.

Differential metabolites (DMs) between the Control and Model groups and between the Model and JG groups revealed a comprehensive understanding of endogenous metabolite changes in neuropathic pain rats. DMs were identified according to max fold change >2, the VIP > 1.0, CV > 30, and *p* < 0.05. Referring to the literature reports and searching in the online HMDB database, there were 113 up-regulated metabolites and 8 down-regulated in the Model group compared with the Control group. Meanwhile, compared with the Model group, 2 up-regulated metabolites and 67 down-regulated metabolites were found in the JG-treated group. There were 39 common DMs that appeared in each group, as shown in Figure 7A. They were selected for biomarkers that may be related to the analgesic effect of JG on neuropathic pain. Their detailed information is listed in Table 4. The corresponding heatmap in Figure 7B provides a visual representation of the change among the three groups. These results suggested that JG can regulate metabolic disorders through the key metabolites analyzed above.

#### 2.4.2. Metabolic Pathway Enrichment Analysis

**Figure 8:**
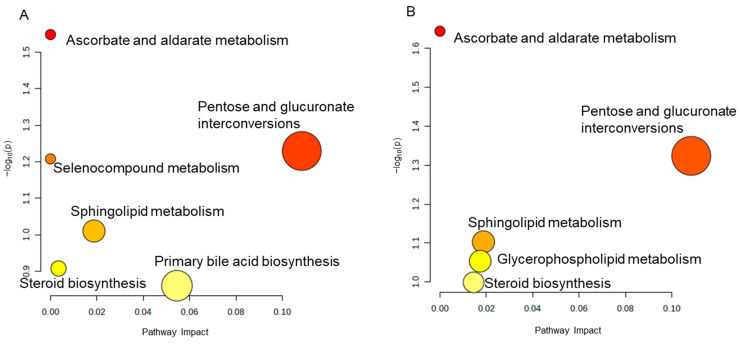
Metabolic pathway analysis of differential metabolites (DMs). (**A**) Enriched metabolic pathways by Model-DMs. (**B**) Enriched metabolic pathway by JG-DMs.

A pathway enrichment analysis was conducted using Metaboanalyst 5.0 (http://www.metaboanalyst.ca/ (accessed on 22 November 2023)). In the Model group, pentose and glucuronate interconversions, sphingolipid metabolism, and primary bile acid biosynthesis may be affected by PTX, while JG treatment primarily regulated pentose and glucuronate interconversions, sphingolipid metabolism, and glycerophospholipid metabolism in PIPN rats (Figure 8A,B). These results revealed that the potential antinociceptive mechanism of JG is mainly related to the regulation of pentose and glucuronate interconversions and sphingolipid metabolism pathways in metabolic response.

### 2.5. Gut Microbiota Analysis

#### 2.5.1. Gut Microbiota Diversity Variation

**Figure 9:**
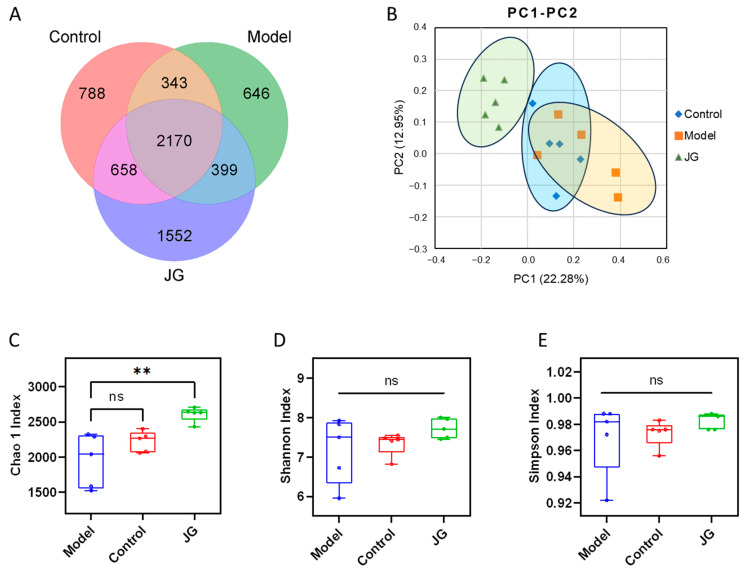
α and β diversity in the cecum samples from the three experimental groups. (**A**) Venn diagram showing the shared and unique OTUs across three experimental groups. (**B**) Principal coordinate analysis (PCoA) ordination plot for the three groups. (**C**) Chao1 index. (**D**) Shannon index. (**E**) Simpson index. ** *p* < 0.01, vs. Model group. ns, *p* > 0.05.

The overall structural changes of gut microbiota were determined by analysis of the 16S rRNA gene sequences of microbial samples isolated from the cecum of the Control, Model, and JG groups. The Venn diagram shows the OTUs shared between groups and those that are unique to each group (Figure 9A). Treatment with the JG extract increased the number of OTUs.

The α-diversity analysis reflects the abundance and diversity of a single sample and includes the Chao1, Shannon, and Simpson indexes. As shown in Figure 9C–E, the Chao1 index of the JG group was higher than the Control and Model groups, indicating a higher species abundance, while the Shannon and Simpson indexes are similar among groups, indicating a similar community diversity. The β-diversity reflects the presence of significant differences in the microbial communities between the samples. In this study, principal coordinate analysis (PCoA) was used to identify differences in species diversity. As shown in Figure 9B, the samples of the three groups were distinct, indicating that the species structure is quite different.

#### 2.5.2. Analysis of Species Abundance

**Figure 10:**
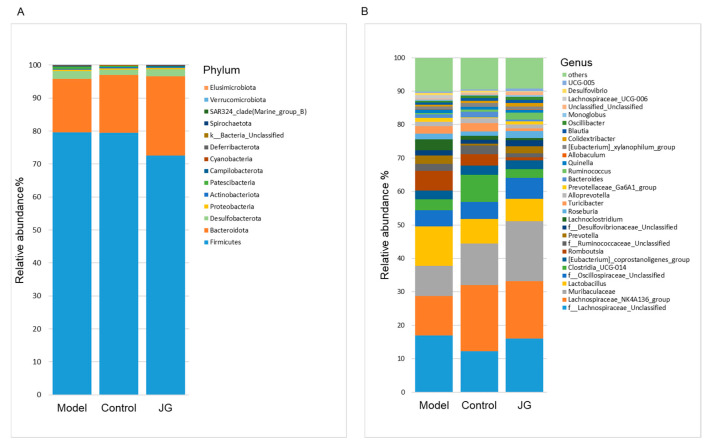
Gut microbiota compositions at the phylum (**A**) and genus (**B**) levels for the three experimental groups.

**Figure 11:**
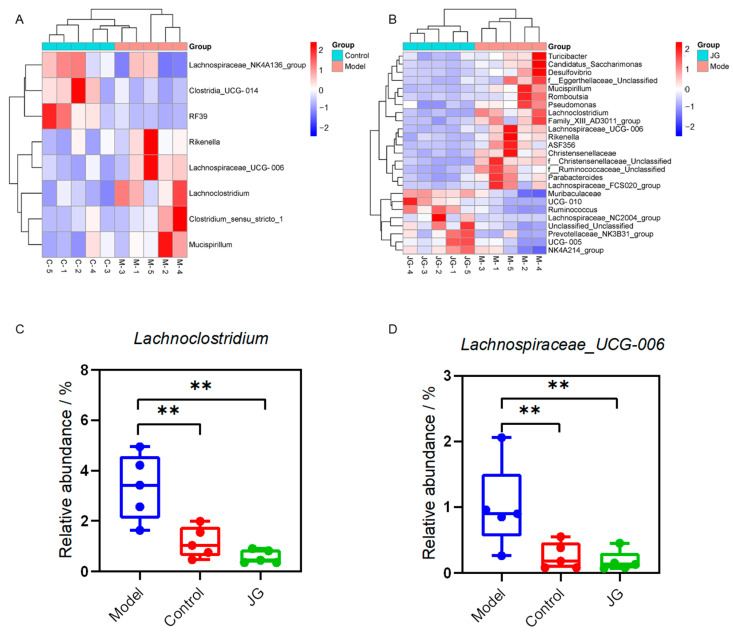
PTX and JG treatment altered the composition of gut microbiota at the genus level. (**A**) Heatmap indicating the significantly changed microbiota between the Model group and Control group. (**B**) Heatmap indicating the significantly changed microbiota between the JG treatment group and the Model group. (**C**) JG reversed the abundance of *Lachnoclostridium* changed in the Model group. (**D**) JG reversed the abundance of *Lachnospiraceae_UCG-006* changed in the Model group. ** *p* < 0.01, vs. Model group.

Furthermore, we compared the relative abundance of the predominant taxa in the three groups. At the phylum level, all samples shared similar community structures. *Firmicutes* and *Bacteroidetes* were the dominant phyla, representing more than 95% of the relative abundance (Figure 10A). At the genus level, each group of samples included mostly *Lachnospiraceae*, *Muribaculaceae*, *Lactobacillus*, *Oscillospiraceae*, *Clostridia*, etc. The relative abundance of different species varied among the three groups (Figure 10B). More precisely, the abundance of *Clostridium_sensu_stricto_1*, *Mucispirillum*, *Rikenella Lachnoclostridium,* and *Lachnospiraceae_UCG-006* increased significantly, while *RF39*, *Lachnospiraceae_NK4A136_group*, and *Clostridia_UCG-014* decreased significantly in the Model group compared with the Control group (Figure 11A). We can find more changes in the JG group when compared with the Model group (Figure 11B). What is more, the changes of *Lachnoclostridium* and *Lachnospiraceae_UCG-006* of the Model group were reversed by JG treatment significantly (Figure 11C,D).

## 3. Discussion

Phytocannabinoids in hemp mainly consist of abundant CBDA, CBD, CBDVA, and CBDV and little THC, which is different from marijuana rich in THC. In this study, JG mainly consisting of phytocannabinoids was enriched from hemp through supercritical CO_2_ extraction, decarboxylation, and the alcohol precipitation process. The content of CBD and THC in JG was 30.1% and 2.42%, respectively, with a ratio of 13:1, which is similar to that of reported cannabis extracts used to study the antinociceptive effect in CCI and the streptozotocin-induced diabetic neuropathy model with a ratio of 16:1 [18,19]. According to our study, the cannabinoid components of JG also showed analgesic effects in the PIPN model.

In the present study, PTX-treated rats exhibited mechanical allodynia and thermal hyperalgesia from day 4 and day 8, respectively, and JG administration reversed it significantly on day 10, so biological samples for omics research were collected on day 11 to make sure there was a significant difference among groups in behavioristics.

Based on RNA-sequence analysis, changes in response to heat, temperature homeostasis upon Model-DEGs GO enrichment analysis, and inflammatory mediator regulation of TRP channels upon KEGG pathway analysis were inconsistent with changes in animal behavioristics. TRP channels are a group of ion channel receptors located in the cell membrane and form a large channel group consisting of 6 subfamilies and 28 channels in humans [26]. Many of them were observed to be temperature-sensitive and involved in chemotherapy-induced neuropathic pain [27].

Moreover, the transcriptome changes of the Model group also reflect the possible mechanism of PIPN, mainly related to ion transmembrane transport, cellular calcium ion homeostasis, DNA-binding transcription activator activity, transmembrane signaling receptor activity, neuroactive ligand–receptor interaction, inflammatory mediator regulation of TRP channels, etc. These results were consistent with a previous DRG transcriptomic analysis result, suggesting that neuroactive ligand–receptor interaction and cytokine–cytokine receptor interaction are majorly involved in the sensory neurons of rats with PIPN [28]. Moreover, previous studies have reported that lncRNA/mRNA expression profiles in spinal cord dorsal horn showed the most significantly enriched pathways, including immune/inflammatory responses and neurotrophin signaling pathways, which are all important mechanisms mediating neuroinflammation, central sensitization, and chronic pain [29].

JG-DEGs reflected the transcriptional regulation of JG on PIPN model rats, which may include the regulation of the disease itself and the normal organism. Enrichment analysis revealed that the JG-DEGs are mainly involved in neuroactive ligand–receptor interaction, circadian rhythm, cellular calcium ion homeostasis, lipid catabolic process, and the serotonin receptor signaling pathway. However, the most enriched pathway circadian rhythm may not be involved in the analgesic effects. There was sufficient evidence to suggest that cannabis use alters circadian rhythms [30,31]. Moreover, the most enriched pathways may vary from experiments, so we mainly analyzed the common DEGs as the key point of the analgesic effect of JG on PIPN rats. As shown in Figure 4E, Cnga3, Fabp4, Fst, Ldhc, Oprd1, Tg, and Trpv4 may be the most important genes regulated by JG to play a critical role in the analgesic effect. The gene Ldhc is mainly involved in metabolic processes, such as pyruvate metabolism and glycolysis/gluconeogenesis. Cnga3 enriched in the cAMP signaling pathway is involved in ion transmembrane transport. Neuroactive ligand–receptor interaction pathways were mainly GPCRs, ion channels, chemokines, and cytokines, which play important roles in the regulation of sensory perception of pain. Fabp4 enriched in the PPAR signaling pathway is mainly related to fatty acid metabolic and transport processes. Follistatin (FST) is an endogenous blocker of the TGF-β signaling pathway. It was demonstrated that it neutralizes TGF-β1’s activity and protein expression, while TGF-β1 is effective in neuropathic treatment by targeting both neurons and glial cells [32]. Transient receptor potential vanilloid type 4 (TRPV4), belonging to the TRP superfamily, is expressed in immune cells, sensory neurons, glial cells, the spinal cord, cortical pyramidal neurons, etc. This channel is associated with inflammatory diseases that affect the central and peripheral nervous system, such as osteoarthritis, atherosclerosis, cancer pain, and neuropathies [33]. Several studies have shown that cannabinoids are activators of TPR channels [34,35]. These DEGs screened in this study may play a critical role in the regulation of protein expression in PIPN, but they need to be further verified, and future in-depth studies are required.

From the transcriptome, several metabolic-related pathways are enriched. To deeply investigate the mechanisms underlying the effects of JG on PIPN rats, we carried out serum untargeted metabolomic analysis. In total, 118 significantly differential metabolites were identified in the PIPN rats, most of which belonged to lipids. The result showed that PTX primarily disturbed glycerophospholipid metabolism, which was in accord with the published literature [36]. During the process of JG treatment, 70 metabolites were changed compared with the Model group. JG treatment rebalanced the alteration of most metabolites disturbed by paclitaxel. The 39 common DMs played a critical role in attenuating mechanical allodynia and thermal hyperalgesia primarily by regulating pentose and glucuronate interconversions and sphingolipid metabolism pathways.

Gut microbiota serves as the intersection of immune, neural, endocrine, and metabolic signaling pathways and has become an intense focus of research [37]. Several studies have demonstrated that gut microbiota can modulate chemotherapy-induced neuropathic pain [38,39], even that the gut bacteria, rather than host genetics or physiology, are the primary determinants of PTX-induced pain based on a comparative study of paclitaxel responses in B6 and 129 germ-free reciprocally transplanted mice [40]. The study by Loman et al. revealed the relationship among behavior, central and peripheral immune activation, and microbiota in PTX-treated mice [41]. In our study, the three experimental groups shared a similar community diversity. The significantly changed microbiota in the Model group shown in Figure 11A, including increased *Clostridium_sensu_stricto_1*, *Mucispirillum*, *Rikenella Lachnoclostridium,* and *Lachnospiraceae_UCG-006* and decreased *RF39*, *Lachnospiraceae_NK4A136_group*, and *Clostridia_UCG-014,* might exert certain effects on the development of PIPN. Though *Lachnoclostridium* is an important bacterium that produces SCFAs and can exhibit anti-inflammatory effects in the body, it is usually a risk factor for the development of some diseases [42]. It is important to note that the pathogenic or beneficial function of several gut microbes may be strain- and context-specific [43]. *Clostridium_sensu_stricto_1* is an intestinal pathogenic bacteria associated with a higher risk of necrotizing enterocolitis. As reported by Abdelbary MMH, etc., the inflammatory bowel disease patients had a significantly higher abundance of *Clostridium sensu stricto 1* [44]. When treated with JG, the abundances of *Lachnoclostridium* and *Lachnospiraceae_UCG-006* were reversed significantly in PIPN rats, which may play an important role in preventing mechanical and thermal hypersensitivity for JG.

Altogether, using omics-based approaches, we obtained an overall understanding of the analgesic mechanism of JG, which may be mainly regulated by the identified 7 key genes, 39 metabolic biomarkers, and 2 bacterial genera. Functional analysis revealed that they were mainly involved in the neuroactive ligand–receptor interaction pathway, PPAR signaling pathway, inflammatory mediator regulation of TRP channels, ion transmembrane transport, sphingolipid metabolism, pentose and glucuronate interconversions, etc.

However, correlation does not imply causation. Collecting data at different time points and from more rats may be conducive to obtaining more specific conclusions. In addition, experiments are still needed to further verify and explore the bioinformatics prediction results.

## 4. Materials and Methods

### 4.1. Materials and Reagents

The plant material utilized in this study (flowers and leaves powder, DM-Y-013) was cultivated hemp (*Cannabis sativa* L.) with THC < 0.3% in dry biomass provided by Yunnan Hemp Valley Biological Technology Co., Ltd. (Qujing, China). Paclitaxel (PTX) (30 mg, 5 mL, 22,102,911) was purchased from Yangtze River Pharmaceutical Group Co., Ltd. (Taizhou, China). Gabapentin (0.1 g in each capsule, JB220405) was purchased from Jiangsu Enhua Pharmaceutical Co., Ltd. (Xuzhou, China). Reference compounds cannabidiol (CBD, 1.0mg/mL in methanol, FE01271601) and Δ-9-tetrahydrocannabinol (Δ9-THC, 1.0 mg/mL in methanol, FE08221804) were purchased from Beijing Finger Eran Technology Co., Ltd. (Beijing, China). Formic acid (mass spectrometry grade, 50 mL, 186,260) was from Thermo Fisher (Waltham, MA, USA). Carbon dioxide (food grade, 40 L/bottle) was provided by Lvmin Gas Co., Ltd. (Shanghai, China). Acetonitrile (LC-MS grade) was bought from Shanghai Adamas Reagent Co., Ltd. (Shanghai, China). All of the other chemicals were of analytical purity.

### 4.2. Preparation and Analysis of Cannabis sativa L. Extract (JG)

The extraction was carried out on the laboratory-made supercritical fluid carbon dioxide extraction (SFCE) system consisting of a CO_2_ reservoir, cooling bath, air compressor, air-driven CO_2_ pump, heating bath, extraction kettle, separator vessel, and flow meter (GKSFE230-40-(5 + 5) L, Jiangsu gaoke Pharmaceutical Equipment Co., Ltd., Nantong, China). The *Cannabis sativa* L. plant material (660 g, flowers and leaves powder) was poured into the extraction kettle and reflux extracted for 2 h under the condition of 32 MPa, 55 °C, and the flow rate of CO_2_ was 30 L/h [20]. Then, the SFCE extraction was heated under 125 °C for 5h for decarboxylation and precipitated with 85% ethanol with a ratio of 1:10 for 2 h to remove impurities of wax and pigment. The extraction conditions were determined based on references and by the yield of CBD upon several experiments performed [45]. Then, the supernatant was concentrated to obtain the *Cannabis sativa* L. *Extract* (JG) rich in phytocannabinoids. Then, the composite profile was analyzed by UPLC-QTOF MS and HPLC.

A Waters Acquity UPLC system with a Waters ACQUITY UPLC HSS T3 analytical column (2.1 mm × 150 mm, 1.8 µm, Waters, Milford, MA, USA) connected to a Waters Xevo G2-XS QTOF mass spectrometer (Waters Co., Ltd., Milford, MA, USA) was used to perform chromatographic separations and mass spectrometry detections via electrospray ionization interface. The mobile phase consisted of water with 0.1% formic acid (A) and acetonitrile (B) at 0.3 mL/min, and the gradient program was as follows: 10–65% B over 0–10 min, 65–95% B over 10–60 min, 95% B over 60–65 min, 95–10% B over 65–66 min, and 10%B over 66–70 min. Main compounds were identified based on references [23,46]. Mass spectrometry acquisition conditions were as follows: full-scan mass range, *m*/*z* 50–1500 Da; drying gas (N2) flow rate, 800 L/h; drying gas temperature, 400 °C; source temperature, 120 °C; sample cone voltage, 40 V; cone gas flow, 100 L/h.

The content of CBD, the most abundant phytocannabinoid in JG, was further determined with a commercial reference using a Thermo UPLC system (Waltham, MA, USA) with a UV detector on 220 nm [47].

### 4.3. Animals and Treatment

Healthy male Wistar rats (SPF, 6~8 weeks old) were purchased from Shanghai SLAC Laboratory Animal Co., Ltd. (Certificate No. SCXK, 2022-0004, Shanghai, China). The rats were allowed to adapt to the environment for >1 week before the experiments. They were arranged in groups of 10 and housed in a plastic cage under a 12 h dark/12 h light cycle (25 °C; 55 ± 5% RH) with standard chow and water ad libitum. All animals were finally euthanized by cervical dislocation in this study. All procedures involving rats were approved by the Animal Ethics Committee of the Centre for Pharmacological Evaluation and Research in Shanghai Institute of Pharmaceutical Industry.

To evaluate the anti-neuropathic pain effect of JG in vivo, the PTX-induced neuropathic rat model was established [48]. Forty rats were randomly divided into 5 groups with 8 animals in each group as follows: Control group (blank control, vehicle, i.p.), Model group (neuropathic pain model, vehicle, i.p.), Gabapentin group (positive drug control, gabapentin 100 mg/kg, i.g.), JG-L group (JG 15 mg/kg, i.p), and JG-H group (JG 30 mg/kg, i.p.). Except for the Control group, rats were administered PTX 2 mg/kg daily on days 1, 3, 5, and 7 to induce neuropathic pain, and drugs on days 2, 4, 6, 8, 9, and 10. Mechanical and thermal hyperalgesia were measured on days 0, 4, 8, and 10. JG was diluted with 3% tween 80 saline solution. Rats in the Control group were fed with saline vehicle. Blood was collected by removing the eyeballs after the last administration and behavioral tests on day 10. Serum was collected by centrifugation at 5000 rpm for 10 min at 4 °C. After the animals were sacrificed by cervical dislocation, cecum contents and the lumbar L3~L6 spinal cord were collected, and biochemical and molecular parameters were examined.

### 4.4. Behavioral Tests

Mechanical and thermal hyperalgesia were measured by experimenters blinded to the treatments. Rats were placed in transparent boxes (20 × 20 × 10 cm) for 20 min until they stopped obvious exploratory behaviors and were in a quiet state. Baseline responses to mechanical and thermal stimuli were recorded one day before the start of drug administration. Subsequent behavioral tests were performed on days 4, 8, and 10 after administration.

#### 4.4.1. Mechanical Allodynia

For assessment of mechanical allodynia, von Frey filaments ranging from 4 to 60 g bending force were applied to the plantar skin of the right hind paw, and each application was held for 6 s, using the up–down method to determine the paw withdrawal threshold [48].

#### 4.4.2. Thermal Hyperalgesia

Thermal hyperalgesia was evaluated using the Hargreaves test by measuring the thermal withdrawal latency of the foot to heat stimulation, in which animals were acclimatized to plexiglass enclosures positioned on a prewarmed glass plate [48]. A radiant heat source (IITC Life Science Plantar Test), directed at the plantar surface of the hind paw, was used to elicit paw withdrawal. The stimulus shut off when the hind paw moved (or after 25 s to prevent tissue damage). Each animal was measured 3 times with a five-minute interval. The results of thermal withdrawal latency represent the mean values of ipsilateral feet.

### 4.5. Enzyme Linked Immunosorbent Assay

The contents of inflammatory cytokines in the serum of rats were determined using ELISA kits, according to the manufacturer’s instructions, including a rat tumor necrosis factor-α kit (TNF-α, ER20497M) and a rat Interleukin-1 Beta kit (IL-1β, ER20275M) (Shanghai Weiao Biotechnology Co., Ltd., Shanghai, China).

### 4.6. Transcriptomic Analysis of Spinal Cord

#### 4.6.1. RNA Extraction and Sequencing

Library preparation and transcriptome sequencing were performed at Suzhou Jinweizhi Biotechnology Co., Ltd. (Suzhou, China). In brief, total RNA was extracted from spinal cord samples using a MagZol Reagent (RNA Reagent kit, AJ15RK01, Guangzhou Magen Biotechnology Co., Ltd., Guangzhou, China). The concentration of RNA was measured using a Nanodrop 2000 spectrophotometer (Thermo Fisher Scientific, Waltham, MA, USA). A total of 1 μg total RNA was used for following library preparation. The poly(A) mRNA isolation was performed using Oligo(dT) beads. The mRNA fragmentation was performed using divalent cations and high temperatures. Priming was performed using random primers. The purified double-stranded cDNA was then treated to repair both ends and add a dA-tailing in one reaction, followed by a T-A ligation to add adaptors to both ends. Each sample was then amplified by PCR using P5 and P7 primers, and the PCR products were validated. Then, libraries with different indexes were multiplexed and loaded on an Illumina HiSeq instrument for sequencing using a 2 × 150 paired-end (PE) configuration according to the manufacturer’s instructions [49].

#### 4.6.2. Data Processing and Bioinformatics Analysis

The DESeq2 Bioconductor package was used to analyze differential expression genes (DEGs) [50]. The default screening threshold for DEGs was |log 2 FC| > 1 and Padj < 0.05. GOSeq (v1.34.1) was used to identify Gene Ontology (GO) terms that annotate a list of enriched genes with a significant padj ≤ 0.05. KEGG (Kyoto Encyclopedia of Genes and Genomes) (http://en.wikipedia.org/wiki/KEGG (accessed on 5 August 2023)) was used to enrich pathways for significantly differential expression genes.

### 4.7. Untargeted Metabolomic Analysis of Serum Samples

#### 4.7.1. Sample Preparation and UPLC-Q-TOF/MS Metabolomics Analysis

The stored plasma in the Control, Model, and JG-H groups was remelted, and 200 µL serum of each sample was mixed with 600 µL precooled methanol, following vortex oscillation for 60 s. The mixture was placed at −20 °C for 30 min and centrifuged at 14,000× *g* for 10 min. Then, about 600 µL of supernatant was dried in a vacuum environment and dissolved by a 100 µL methanol/water mixture (1:1), followed by vortex oscillation. After centrifugation at 14,000× *g* for 10 min at 4 °C, the upper phase of the sample was collected, from which 20 µL of each sample was mixed and used as a quality control sample (QC). All samples were subjected to UPLC-Q-TOF/MS for analysis.

The UPLC-Q-TOF/MS system was the same as described in Section 4.2. The column temperature was 40 °C. The mobile phase consisted of water with 0.1% formic acid (A) and acetonitrile (B) at 0.3 mL/min, and the gradient program was as follows: 5% B over 0–1 min, 5–68% B over 1–14 min, 68–80% B over 14–24 min, 80–90% B over 24–28 min, 90–100% B over 28–30 min, 100–5% B over 30–30.5 min, and 5% B over 30.5–35 min. Mass spectrometric conditions included both positive and negative ion modes. The QC samples were used every eight samples to evaluate the stability during sequence analysis.

#### 4.7.2. Data Processing and Multivariate Analysis

All mass spectral data of the samples were imported into Progenesis QI 3.0.3 (Waters Corporation) for data processing, including peak alignment, peak extraction, and normalization [51]. Principal component analysis (PCA), and partial least squares discriminant analysis (PLS-DA) were used to analyze the trend of metabolites. The potential differential metabolites were screened according to VIP > 1, MAX fold change > 2, minimum CV > 30 and *p* < 0.05 rules. The obtained differential metabolites were retrieved and confirmed in the Human Metabolome Database (HMDB) (https://hmdb.ca/ (accessed on 2 August 2023)). Based on the Kyoto Encyclopedia of Genes and Genomes (KEGG) database, the related metabolic pathways of the differential metabolites were determined.

### 4.8. Gut Microbiota Analysis of Cecum Content

Library preparation and 16S rDNA sequencing were performed at Suzhou Jinweizhi Biotechnology Co., Ltd. (Suzhou, China). In brief, total genome DNA from samples was extracted with HiPure Stool DNA Kit (lot, DJF25-01, Guangzhou Magen Biotechnology Co., Ltd., Guangzhou, China) according to the manufacturer’s protocols. DNA concentration was monitored by a Qubit^®^ dsDNA HS Assay Kit (Guangzhou Magen Biotechnology Co., Ltd., Guangzhou, China). A total of 20–50 ng of DNA was used to generate amplicons that cover V3 and V4 hypervariable regions of the 16S rRNA gene of bacteria. The forward primer contains the sequence ‘CCTACGGRRBGCASCAGKVRVGAAT’, and the reverse primer contains the sequence ‘GGACTACNVGGGTWTCTAATCC’. The concentration is detected by a microplate reader (Tecan, Infinite 200 Pro), and the fragment size is detected by 1.5% agarose gel electrophoresis, which is expected at ~600 bp. Next-generation sequencing was conducted on an Illumina Miseq/Novaseq Platform (Illumina, San Diego, CA, USA) at the company. Automated cluster generation and 250/300 paired-end sequencing with dual reads were performed according to the manufacturer’s instructions. All quality-filtered sequencing reads were then clustered into operational taxonomic units (OTUs) with a threshold of 97% pairwise identity using the VSEARCH clustering (1.9.6) sequence. Then, the RDP classifier (Ribosomal Database Program) Bayesian algorithm was used to annotate the taxonomic information of OTUs for each representative sequence. The OTU abundance information was normalized utilizing a standard sequence number corresponding to the sample with the least sequences. Subsequent analyses were all performed based on these normalized output data.

### 4.9. Statistical Analysis

SPSS 22.0 statistical software was used to analyze the data, expressed as mean ± standard deviation (SD), and a one-way ANOVA test was used for data analysis, with *p* < 0.05 determining statistical significance. For mapping, GraphPad Prism 9.2 software was used.

## 5. Conclusions

In conclusion, the antinociceptive effects and mechanism of *Cannabis sativa* L. extract rich in cannabinoids in PIPN rats were evaluated by using pharmacological methods integrated with transcriptomic analysis, metabolomic analysis, and gut microbiota analysis. *Cannabis sativa* L. extract effectively alleviated neuropathic pain induced by PTX, mainly by the identified 7 key genes, 39 metabolic biomarkers, and 2 bacterial genera. Related pathways may be involved in the inflammatory response, regulating neuroactive ligand–receptor interaction pathway, PPAR signaling pathway, inflammatory mediator regulation of TRP channels, glycerophospholipid metabolism, pentose and glucuronate interconversions, etc. Our study provides novel insights into the functional interactions of *Cannabis sativa* L. extract on PIPN, which offers key information for new strategies in PIPN treatment and provides a reference for the medicinal development of hemp. As mechanism analysis was based on omics bid data, experiments are still needed to further verify the results.

## Figures and Tables

**Table 1:** Compounds in hemp extract (JG) identified using the UPLC-QTOF/MS method.

No.	RT (min)	Tentative Identification	Formula	*m*/*z*	Molecular Ion Peak	Adducts (Positive)	Adducts (Negative)
1	8.10	Cannabispiradienone [2]	C_15_H_14_O_3_	242	243.1405 [M + H]^+^, 241.9238 [M − H]^−^		227.1516
2	8.61	Cannabispireone [2,23]	C_15_H_16_O_3_	244	245.1581 [M + H]^+^, 243.1261 [M − H]^−^		229.1683
3	8.91	Cannabispiran [2,23]	C_15_H_18_O_3_	246	247.1710 [M + H]^+^, 245.1418, [M − H]^−^	189.1302	161.0751
4	9.97	CBE-C3 [23]	C_19_H_26_O_3_	302	303.1593 [M + H]^+^, 301.1361 [M − H]^−^	271.1359, 257.1559, 243.1405	286.1120, 271.0879, 243.9218
5	10.15	Cannabichromanone-D [2]	C_21_H_28_O_3_	328	329.2095 [M + H]^+^, 373.2010 [M + 45]^−^, 327.1910 [M − H]^−^	301.1425, 289.2181, 273.1477	293.2042, 236.1302
6	10.48	CBT-C3 [2]	C_19_H_26_O_4_	318	319.2248 [M + H]^+^, 317.2080 [M − H]^−^	301.2136, 285.1125	299.1954, 281.1802, 257.1778, 163.0930
7	10.65	CBGAM [23]	C_23_H_34_O_4_	374	749.4123 [2M + Na]^+^, 397.2253 [M + Na]^+^, 375.2461 [M + H]^+^, 373.2367 [M − H]^−^	357.2377, 339.2273, 275.1671	329.2462, 311.2404
8	11.18	CBT-C5 [23]	C_21_H_30_O_4_	346	347.2566 [M + H]^+^, 345.2388 [M − H]^−^	329.2466, 311.2323, 279.2690, 231.1763	327.2280, 313.2704, 289.1752, 271.1621
9	11.36	CBR [23]	C_21_H_32_O_4_	348	371.2651 [M + Na]^+^, 349.2691 [M + H]^+^, 347.2583 [M − H]^−^	327.1929, 313.499, 297.2774, 279.2690	329.2462, 311.2513, 295.2238
10	11.50	CBCV [23,24]	C_19_H_26_O_2_	286	287.2380 [M + H]^+^, 285.2121 [M − H]^−^	201.2038	191.1242
11	11.79	Cannflavin B [2]	C_21_H_20_O_6_	368	369.1666 [M + H]^+^, 367.1573 [M − H]^−^	313.1050, 291.2330, 193.1616	331.2632
12	12.08	CBEAB-C3 [2]	C_20_H_26_O_5_	346	369.1666 [M + Na]^+^, 347.1804 [M + H]^+^, 345.2083 [M − H]^−^	313.2499, 201.2136, 715.2702	311.2332, 329.2462, 191.1242
13	12.69	CBT-OEt-C3 [2]	C_21_H_30_O_4_	346	347.2566 [M + H]^+^, 345.2388 [M − H]^−^	329.2429, 277.2551	309.2170, 285.2121, 279.1648, 191.1242
14	13.25	CBDVA [23,24]	C_20_H_26_O_4_	330	331.2621 [M + H]^+^, 329.2425 [M − H]^−^	313.2499, 275.2383	325.2123, 309.2350
15	13.83	4-Acetoxy-CBC [2]	C_23_H_32_O_4_	372	373.2312 [M + H]^+^, 371.2218 [M − H]^−^	355.2217, 331.2323, 313.2173, 279.2690	329.2091, 311.1971, 295.2555, 277.2453
16	14.09	Epoxycannabigerol [2]	C_21_H_32_O_3_	332	333.2763 [M + H]^+^, 331.2595 [M − H]^−^	315.2668, 279.2690	325.2123, 295.2590, 313.2487
17	14.54	CBDV [23,24]	C_19_H_26_O_2_	286	287.2380 [M + H]^+^, 285.2121 [M − H]^−^	231.1763	217.1443
18	14.66	Cannabimovone [2]	C_21_H_30_O_4_	346	347.2566 [M + H]^+^, 345.2388 [M − H]^−^	329.2466	293.2393
19	15.60	CBTT [2]	C_21_H_30_O_5_	362	363.2459 [M + H]^+^, 361.2252 [M − H]^−^	345.2386, 327.2299,	
20	16.10	CBGM [23]	C_22_H_34_O_2_	330	331.2621 [M + H]^+^, 329.2425 [M − H]^−^	313.2499	311.2079, 243.9218
21	16.79	CBND-C3 [23]	C_19_H_22_O_2_	282	283.205 [M + H]^+^, 281.1802 [M − H]^−^	271.2066	243.9218, 311.2007
22	17.07	CBDA [23,24]	C_22_H_30_O_4_	358	341.2466 [M + H − H_2_O]^+^, 357.2701 [M − H]^−^	329.2466, 283.2085	311.2043, 281.1802
23	17.20	Canflavin C [2]	C_26_H_28_O_6_	436	437.2217 [M + H]^+^, 435.2238 [M − H]^−^	383.2517, 359.2556, 313.1050	399.2595, 327.2280, 295.2590, 243.9218
24	18.15	CBG [23,24]	C_21_H_32_O_2_	316	317.2828 [M + H]^+^, 315.2634 [M − H]^−^	287.238	
25	18.63	CBD [23,24]	C_21_H_30_O_2_	314	315.2668 [M + H]^+^, 313.2451 [M − H]^−^	259.2076	313.2270, 245.1771
26	19.45	CBCN-C5 [2]	C_20_H_28_O_4_	332	333.2763 [M + H]^+^, 331.2595 [M − H]^−^	315.2668, 259.2076	325.2160, 293.2077, 243.9218
27	22.78	CBN [23,24]	C_21_H_26_O_2_	310	311.2359 [M + H]^+^, 309.2134 [M − H]^−^	293.2269	277.2453
28	24.63	CBL [23,24]	C_21_H_30_O_2_	314	315.2668 [M + H]^+^		
29	25.37	CBDM [2]	C_22_H_32_O_2_	328	329.2466 [M + H]^+^, 327.2280 [M − H]^−^	315.2668	273.1755, 243.9218
30	26.02	CBC [23,24]	C_21_H_30_O_2_	314	315.2688 [M + H]^+^		
31	27.57	Δ8-THC [23]	C_21_H_30_O_2_	314	315.2668 [M + H]^+^		279.2572, 243.2218
32	28.52	Δ9-THC [23]	C_21_H_30_O_2_	314	315.2668 [M + H]^+^, 313.2487 [M − H]^−^	259.2076	205.1422, 243.9218
33	32.89	CBT-OEt-C5 [23]	C_23_H_34_O_4_	374	397.4129 [M + Na]^+^, 373.2762 [M − H]^−^	357.2416, 331.2994, 207.1803	329.2834

**Table 2:** Content of main phytocannabinoids in hemp extract (JG) tested by HPLC.

Phytocannabinoid	Abbreviation	Content
Cannabidiol	CBD	30.10% ^1^
Cannabidiolic acid	CBDA	0.90% ^2^
Cannabidivarin	CBDV	5.74% ^2^
Δ9-tetrahydrocannabinol	Δ9-THC	2.42% ^2^

^1^ Tested by reference substance, ^2^ calculated by relative peak area based on CBD.

**Table 3:** Common differential expression genes (DEGs) between Model-DEGs and JG-DEGs.

Number	JG Regulation	Gene Symbol
41	Down (27)	Ttll6, Wnt2b, Stc2, AABR07010986.1, Cnga3, Igfbp1, U1, Tbr1, Tg, Oprd1, Foxb1, Efcab10, Igfbpl1, Fst, Ldhc, Pifo, Fbxo17, AABR07047835.1, Duox1, AABR07040887.1, 7SK, AC114452.1, AABR07014306.2, LOC103692165, C1ql4, AABR07026044.1, AABR07037451.3
	Up (14)	Trpv4, Fabp4, Prss54, LOC686087, AABR07073391.1, LOC108348453, LOC100911548, Hsh2d, Tmem182, AABR07045058.1, Slc16a3, AABR07017733.1, AABR07070043.2, AABR07063682.1

**Table 4:** A total of 39 common differential metabolites (DMs) related to therapeutical effects of JG on PIPN.

No.	Rt (min)	ID	Formula	*m*/*z*	Description
1	7.88	HMDB39412	C_54_H_88_O_23_	1127.55	Araliasaponin V
2	8.04	HMDB60075	C_15_H_13_I_2_NO_7_S	1254.69	3′,5′-Diiodo-l-thyronine 4′-*O*-sulfate
3	8.04	HMDB35746	C_57_H_92_O_26_	1215.58	Hovenoside D
4	8.12	HMDB04936	C_61_H_109_N_3_O_26_	1298.71	Ganglioside GM2 (d18:1/12:0)
5	8.12	HMDB32780	C_59_H_96_O_27_	1259.60	Jujuboside C
6	8.85	HMDB29513	C_22_H_16_O_15_S	575.01	Myricatin
7	14.40	HMDB11484	C_25_H_46_NO_7_P	502.29	LysoPE(0:0/20:3(11*Z*,14*Z*,17*Z*))
8	14.40	HMDB10387	C_26_H_48_NO_7_P	562.31	LysoPC(18:3(6*Z*,9*Z*,12*Z*))
9	14.40	HMDB30459	C_29_H_40_N_8_O_5_	579.31	Hordatine B
10	14.49	HMDB10397	C_28_H_48_NO_7_P	586.32	LysoPC(20:5(5*Z*,8*Z*,11*Z*,14*Z*,17*Z*))
11	14.49	HMDB35388	C_34_H_48_O_7_	603.31	(24*E*)-3α,15α-Diacetoxy-23-oxo-7,9(11),24-lanostatrien-26-oic acid
12	15.11	HMDB11129	C_23_H_48_NO_7_P	526.32	LysoPE(0:0/18:0)
13	15.14	HMDB11914	C_55_H_102_N_2_O_21_	1107.68	Ganglioside GM3 (d18:0/14:0)
14	15.14	HMDB35292	C_34_H_50_O_7_	605.32	Ganodermic acid P2
15	15.36	HMDB31906	C_37_H_66_O_2_	1084.01	Muridienin 4
16	15.36	HMDB38189	C_31_H_44_O_6_	1023.63	Carindone
17	15.42	HMDB30317	C_28_H_37_NO_9_	1107.49	Petasinoside
18	15.42	HMDB38337	C_38_H_66_O_2_	1108.01	Helianyl octanoate
19	15.42	HMDB41384	C_23_H_20_O_13_	522.13	Luteolin 3′-(4″-acetylglucuronide)
20	15.45	HMDB35310	C_30_H_44_O_8_	1087.59	Ganoderic acid I
21	15.45	HMDB35356	C_56_H_88_O_20_	1063.59	Pithecelloside
22	15.73	HMDB02513	C_30_H_48_O_9_	1103.66	Lithocholate 3-*O*-glucuronide
23	15.73	HMDB10386	C_26_H_50_NO_7_P	1037.67	LysoPC(18:2(9*z*,12*z*))
24	15.76	HMDB10356	C_24_H_32_O_12_S	543.16	Estriol 3-sulfate 16-glucuronide
25	16.04	HMDB29766	C_24_H_40_O_11_	522.29	(3*S*,7*E*,9*R*)-4,7-Megastigmadiene-3,9-diol 9-[apiosyl-(1->6)-glucoside]
26	16.04	HMDB34731	C_27_H_45_NO_2_	454.31	Tomatidine
27	16.14	HMDB01980	C_46_H_65_N_13_O_12_S_2_	1036.41	Vasopressin
28	16.14	HMDB10382	C_24_H_50_NO_7_P	1035.67	LysoPC(16:0)
29	16.19	HMDB10364	C_27_H_38_O_9_	524.30	11-Hydroxyprogesterone 11-glucuronide
30	16.23	HMDB41366	C_29_H_44_O8	1041.61	24,25-Diacetylvulgaroside
31	16.23	HMDB29341	C_27_H_40_N_4_O_4_	991.61	Ceanothine D
32	16.50	HMDB40684	C_37_H_47_NO_4_	614.34	Janthitrem C
33	16.76	HMDB40914	C_56_H_85_N_15_O_12_	1140.64	Mytilus small cardioactive peptide
34	16.88	HMDB30199	C_28_H_44_N_4_O_4_	1045.68	Frangulanine
35	17.69	HMDB13622	C_19_H_36_O_2_	341.27	Nonadeca-10(*Z*)-enoic acid
36	18.24	HMDB11491	C_27_H_54_NO_7_P	536.37	LysoPE(0:0/22:1(13*Z*))
37	18.47	HMDB33428	C_16_H_18_N_2_O	507.28	(+)-Setoclavine
38	19.30	HMDB29328	C_25_H_29_NO_10_	526.17	Gravacridonediol glucoside
39	19.30	HMDB07925	C_44_H_74_NO_8_P	814.48	PC(14:1(9*Z*)/22:6(4*Z*,7*Z*,10*Z*,13*Z*,16*Z*,19*Z*))

## Data Availability

The data presented in this study are available on request from the authors.

## Supplementary Materials

The following supporting information can be downloaded at: https://www.mdpi.com/article/10.3390/molecules29091958/s1, Figure S1: Base peak intensity chromatogram (BPI) of *Cannabis sativa* L. extract (JG) for UPLC-QTOF/MS analysis. Figure S2: Base peak ion (BPI) chromatogram of serum sample for metabolomic analysis. Table S1: Statistics and quality control of transcriptome sequencing data from spinal cord.
