# Supplementary material for: The Potential Antinociceptive Effect and Mechanism of Cannabis sativa L. Extract on Paclitaxel-Induced Neuropathic Pain in Rats Uncovered by Multi-Omics Analysis
Source: Molecules. 2024 Apr 25;29(9):1958. doi: 10.3390/molecules29091958 (PMC11085863; doi:10.3390/molecules29091958)
Supplement: Supplementary file 1 [file molecules-29-01958-s001.zip › molecules-2943367-supplementary.pdf]

Figure S1. Base peak intensity chromatogram (BPI) of *Cannabis sativa* L. extract (JG) for UPLC-QTOF/MS analysis (A) positive ion mode, (B) Negative ion mode.

Figure S2. Base peak ion (BPI) chromatogram of serum sample for metabolome analysis

Table S1. Statistics and quality control of transcriptome sequencing data from spinal cord

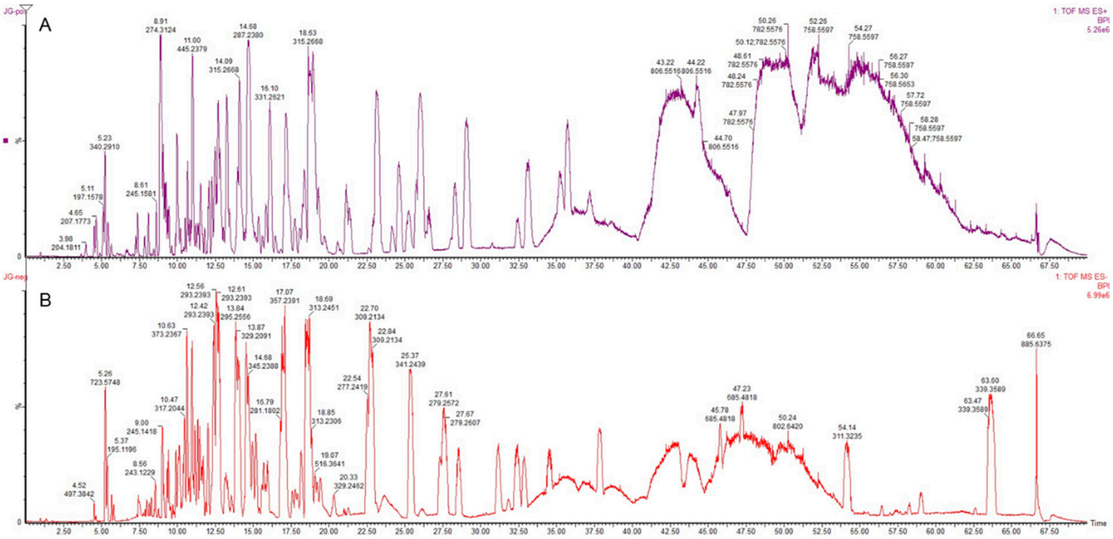

Figure S1. Base peak intensity chromatogram (BPI) of *Cannabis sativa* L. extract (JG) for UPLC-QTOF/MS analysis (A) positive ion mode, (B) Negative ion mode.

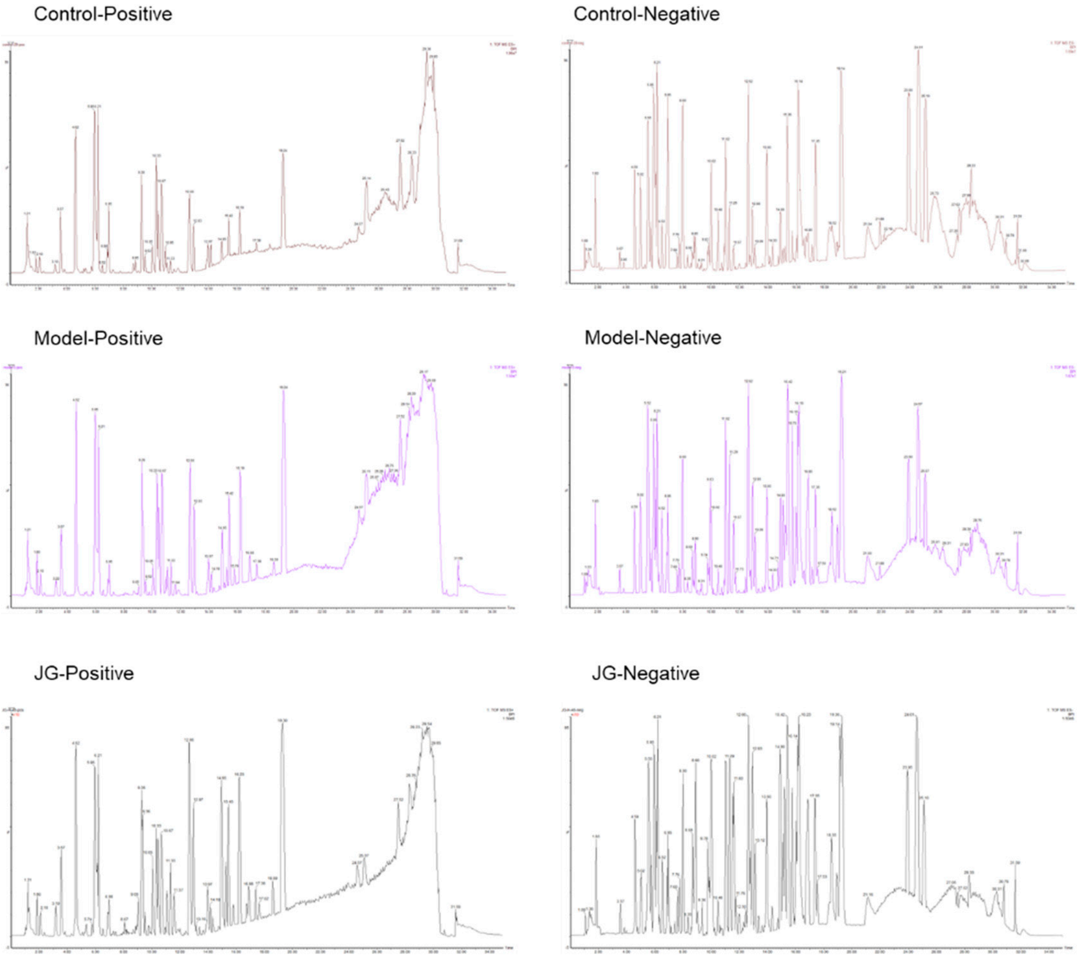

Figure S2. Base peak ion (BPI) chromatogram of serum sample for metabolome Analysis

Table S1. Statistics and quality control of transcriptome sequencing data from spinal cord

Groups	Sample	Raw Reads/million	Clean Reads/million	Q20 (%)	Q30 (%)	GC (%)
Control	C-1	47.53	47.14	98.29	95.02	48.2
	C-2	45.71	45.29	98.15	94.63	47.7
	C-3	50.46	49.98	98.25	94.83	47.65
	C-4	47.51	47.19	98.16	94.7	47.46
Model	M-1	51.92	51.51	98.19	94.74	47.53
	M-2	53.33	52.85	98.19	94.76	47.7
	M-3	43.85	43.45	98.29	95.05	47.88
	M-4	55.57	55.11	98.2	94.75	48.06
JG	JG-1	49.83	49.32	98.21	94.79	47.79
	JG-2	49.41	48.97	98.2	94.75	47.28
	JG-3	54.57	54.04	98.27	94.97	47.57
	JG-4	52.13	51.57	98.22	94.8	47.91
